# Chronic Widespread Spinal Pain (CWSP) Alleviated With Chiropractic Biophysics®: A Case Report With Three-Year Follow-Up

**DOI:** 10.7759/cureus.51620

**Published:** 2024-01-03

**Authors:** Jason W Haas, Miles Fortner, Paul A Oakley, Thomas J Woodham, Deed E Harrison

**Affiliations:** 1 Research, Chiropractic BioPhysics (CBP) NonProfit, Windsor, USA; 2 Chiropractic Biophysics, Western Plains Chiropractic, Gillette, USA; 3 Kinesiology and Health Science, York University, Toronto, CAN; 4 Chiropractic, Chiropractic BioPhysics (CBP) NonProfit, Eagle, USA; 5 Chiropractic, Western Plains Chiropractic, Gillette, USA; 6 Research, Chiropractic BioPhysics (CBP) NonProfit, Eagle, USA

**Keywords:** spine trauma and disease, harrison posterior tangent method, cbp® rehabilitation, abnormal spine alignment, chronic widespread pain (cwsp)

## Abstract

The aim of this case report is to provide clinicians with an option for the treatment of spine pain, spine disorders caused or complicated by abnormal spine alignment, and failed prior interventions for pain and suffering with a conservative protocol. Multi-decade chronic widespread pain (CWSP), low back pain (LBP) headache (HA), and neck pain (NP) cause significant disability and reduced quality of life across all socio-economic and societal categories. Treatment options for decades-old long-term pain with good outcomes are uncommon with non-surgical and surgical interventions. Herein is a single case of positive outcomes with Chiropractic BioPhysics^®^ (CBP^®^)protocol and long-term follow-up. A 60-year-old male with a lifting injury working on a farm at age 12 suffered for decades with LBP, mid-back pain (MBP), NP, HAs, radiculopathy, and poor health-related quality of life (HRQoL). Prior over-the-counter (OTC) medications with diminishing results over 48 years were reported. The patient had multiple abnormal patient-reported outcomes (PROs) as well as measured postural and spine structural abnormalities at the initial assessment. Following 12 treatments, PROs and other measures improved dramatically. Continued brief treatment showed continued progress followed by no treatment beyond continued home exercises and home postural orthoses. All subjective and objective outcome measures improved at one-year follow-up and remained long-term. Improvements in sagittal and coronal postural balance with improved spine alignment, better PROs, and measurably improved HRQoLs were found at one- and three-year follow-ups from the initial evaluation. Chronic NP, LBP, MBP, and extremity pain with altered sensation, loss of function, and failed drug therapy are common across the globe and combined represent the greatest contributors to disability and the global burden of disease (GBD). Economic, efficacious, repeatable, and reliable methods for treating pain will reduce GBD and improve PROs. Larger studies of CBP^®^ methods for multi-decade chronic pain are challenging; however, continued case reports and RCTs for similar conditions are warranted.

## Introduction

We present the individual case of a 60-year-old male who had suffered for 48 years with chronic widespread pain (CWSP), reduced ability, and altered posture with poor spine alignment. The patient was only 12 years old when he was injured in repetitive stress and loading injury (RSI) while lifting, tossing, and stacking hay bales on a farm [1]. The patient remembers suffering from that day in adolescence, into adulthood and continued to have dysfunction and altered range of motion (ROM) as well as pain for 48 years. Chronic low back pain (CLBP), such as this patient’s, is the primary incumbrance of health-related resources and the global burden of disease (GBD) [2].

Treatment options for chronic spine pain after injuries in adolescence followed by multiple decades of suffering prior to successful treatment are not found in the medical literature [3,4]. Due to the near impossible design of such long-term investigations, there are no four-decade or longer studies, randomized controlled clinical trails (RCTs), or others that could be found in patients injured in their early teen years and who suffer from spine pain, loss of range of motion (ROM) and disability. This case demonstrates one of the first long-term measurable improvements in a patient with chronic spine and extremity pain following a specific treatment protocol. This CBP® case study adds to a growing body of multi-modal research evidence demonstrating a reduction in chronic spine pain following a simple, reproducible treatment method [5-10].

CWSP treated with manipulation from traditional chiropractic, or manual and other standard physiological therapeutics (PT) with and/or without over-the-counter medication with long-term improved PROs and measurable outcomes are not found in the literature. Successful long-term surgical and invasive studies showing improved health-related quality of life (HRQoL) measures are not found in 40+ year investigations with long-term follow-up. The purpose of this case is to demonstrate that improving spine alignment, coronal and sagittal balance as well as postural muscular balance following CBP® methods could provide physicians, therapists, and astute clinicians an option to reduce CWSP.

The aim of this study is to provide clinicians with an option to use these conservative methods in patients suffering from pain. CBP® rehabilitation methods use specific Mirror Image® (MI®) exercises, MI® traction, and MI® spinal manipulative therapy (SMT) based upon the average and normal spinal models, digital and machine learning analysis performed by the program PostureRay® (PostureCo™ Inc, Trinity, FL, USA) [11,12]. This reproducible and repeatable radiographic and postural analysis coupled with simple and understandable therapy avenues gives clinicians a conservative, safe, economical, and reliable treatment option for CWSP and CLBP. It may reduce GBD if utilized by physicians treating pain.

## Case presentation

In the fall of 2020, a 60-year-old male (height 180.3cm/weight 118.8kg) suffering from pain and disability in multiple regions. The patient reports headaches and NP frequently with a quadruple visual analog scale (QVAS) average (22/100) [13]. Pain in the upper extremities (UE) focused on the left shoulder most frequently coupled with mid and upper back pain (QVAS 43/100) located between the shoulder blades frequently. He reports constant LBP across the entire upper-, mid-, and lower back. LBP (QVAS 43/100) was the most debilitating complaint. He reported the majority of his spine pain began when he was 12 years old and was caused by lifting and throwing heavy hay bales repeatedly while working on a farm. This chronic pain led him to consume over-the-counter (OTC) medications, such as ibuprofen and acetaminophen, for many years until they were now no longer reducing his pain and causing some GI issues [13,14].

Patient history, orthopedic, neurological, subjective and objective tests and outcome measures were performed. These included the neck disability index (NDI 22/100) headache disability index (HDI 22/100) at initial examination indicating mild disability [15]. The revised Oswestry disability index (RODI 32/50) indicating severe disability due to LBP [16]. The Rand 36 question health status questionnaire demonstrated reduced health perceptions due to pain (57/100), slightly reduced physical function (80/100), overall physical ability significantly reduced due to pain and suffering (50/100), slightly reduced social functioning due to pain (75/100), significant impact of bodily pain on function (58/100) and reduced energy levels due to pain and suffering (50/100). These values are compared to age- and sex-based norms (72, 84, 81, 83, 75, 61), respectively [17].

Compression pain provocation orthopedic tests of the cervical and lumbar spine were found to be positive as well as supine straight leg raising test was positive for both NP and LBP with radicular right-side symptoms into the lower extremity (LE). Hip joint pain provocation tests were also positive for pain in the lumbar spine localized on the right. Palpation of the spine musculature found pain and tenderness in the cervical, thoracic and lumbar spine soft tissues. Additional questioning revealed sexual dysfunction, especially in the presence of CLBP.

Postural evaluation found right head translation compared to normal in the coronal plane (-TxH) coupled with right thoracic coronal translation (-TxT), Further postural evaluation found left thoracic transverse plane rotation (-RzT) and left posterior pelvic rotation (+RyP). Sagittal plane abnormalities were significant with forward head posture (FHP) (+TzH), posterior thoracic translation (-TzT), and anterior translation of the pelvis (+TzP) [18,19]. All postures were visualized and measured using the PostureScreen® Mobile (PSM) application. ROM assessed for pain provocation found restriction with pain during lumbar flexion and left lateral flexion. During the ROM evaluation the patient reported a gluteus medius contracture/spasm bilaterally. Deep tendon reflexes (DTRs) in the LE patellar tendon were severely reduced bilaterally (+1/5). Dermatomal testing found hypoesthesia at the level of L4 on the left and L5 on the right.

**Figure 1:**
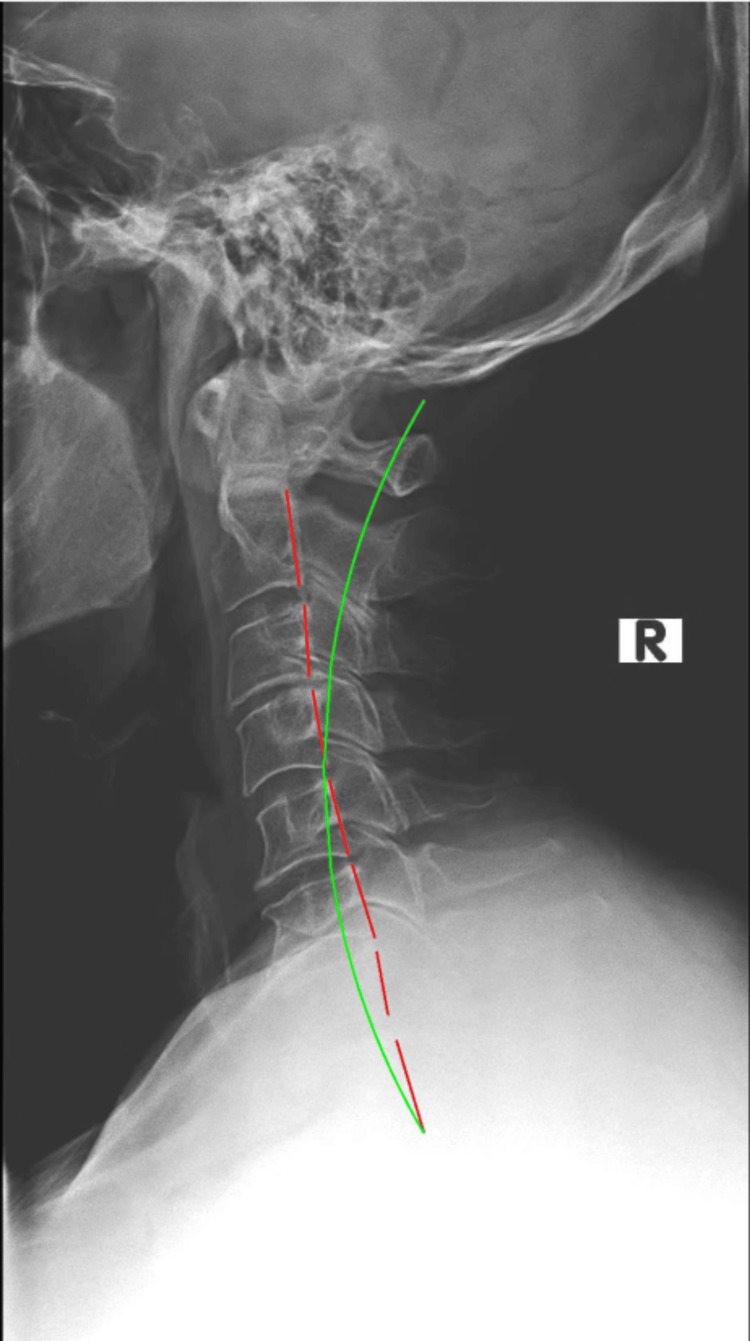
Initial lateral cervical radiograph The red dashed red line demonstrates the Harrison Posterior Tangent Method of mensuration. The green line represents ideal cervical lordosis.

**Figure 2:**
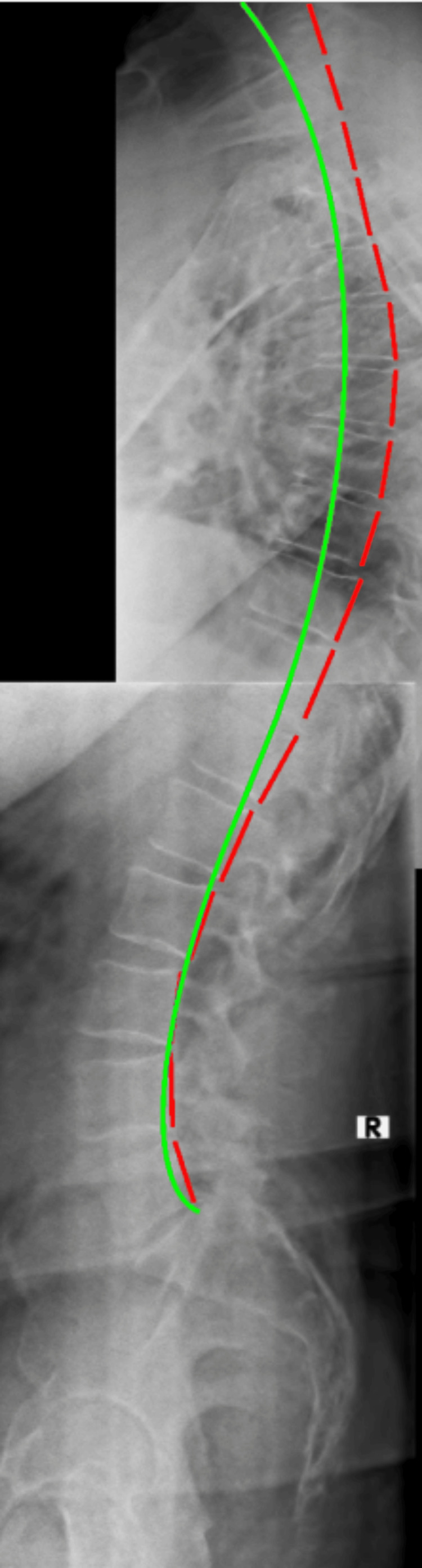
Initial lateral thoracic and lateral lumbar radiographs stitched for sagittal balance analysis The red dashed line measures the posterior tangents of the vertebral bodies using the Harrison Posterior Tangent Method. The green line represents ideal thoracic kyphosis and lumbar lordosis.

Upright radiographs of the full spine were taking in sectional images and were stitched and evaluated with PostureRay®. The assessment measures global and regional structural and spinal abnormal alignment and compares the measured values to normative data and prior spine models. Standard error of measurement using this system is very small (<2°) and the method is repeatable and reliable [20, 21]. Absolute rotation angle (ARAs) of the cervical spine found significant loss of normal lordosis (-2.6°/34°-41°) (Figure 1); patient value/average-ideal) with large anterior head translation (AHT) (24.6mm/0mm). Thoracic ARA was normal from T1-T12 (44.3°/44.0°); however, posterior thoracic translation from T1-T12 was significant (28.8mm/0mm). Lumbar spine biomechanical analysis found well-maintained lordosis; however, there were some degenerative changes at the endplates of L4/L5 and L5/S1 with apparent osteoarthritis as well as loss of disc height (Figure 2). Overall, some degenerative disc changes were found at C3/C4, C5/C6 and C8/C7; T7/T8 and T8/T9; L3/L4 and L5/S1.

**Figure 3:**
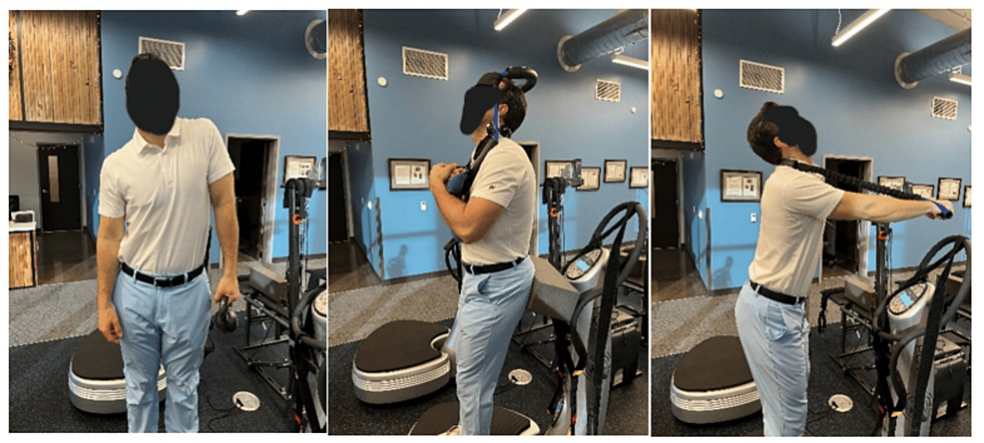
Mirror Image® MI® exercises were performed using whole body vibration (WBV) on the Power Plate® Example patient exercises performed by author (TJW) included thoracic z-axis rotation exercise (left), extension and posterior translation of the cervical spine against resistance with the ProLordotic® (right) cervical Circular Traction device (Center).

**Figure 4:**
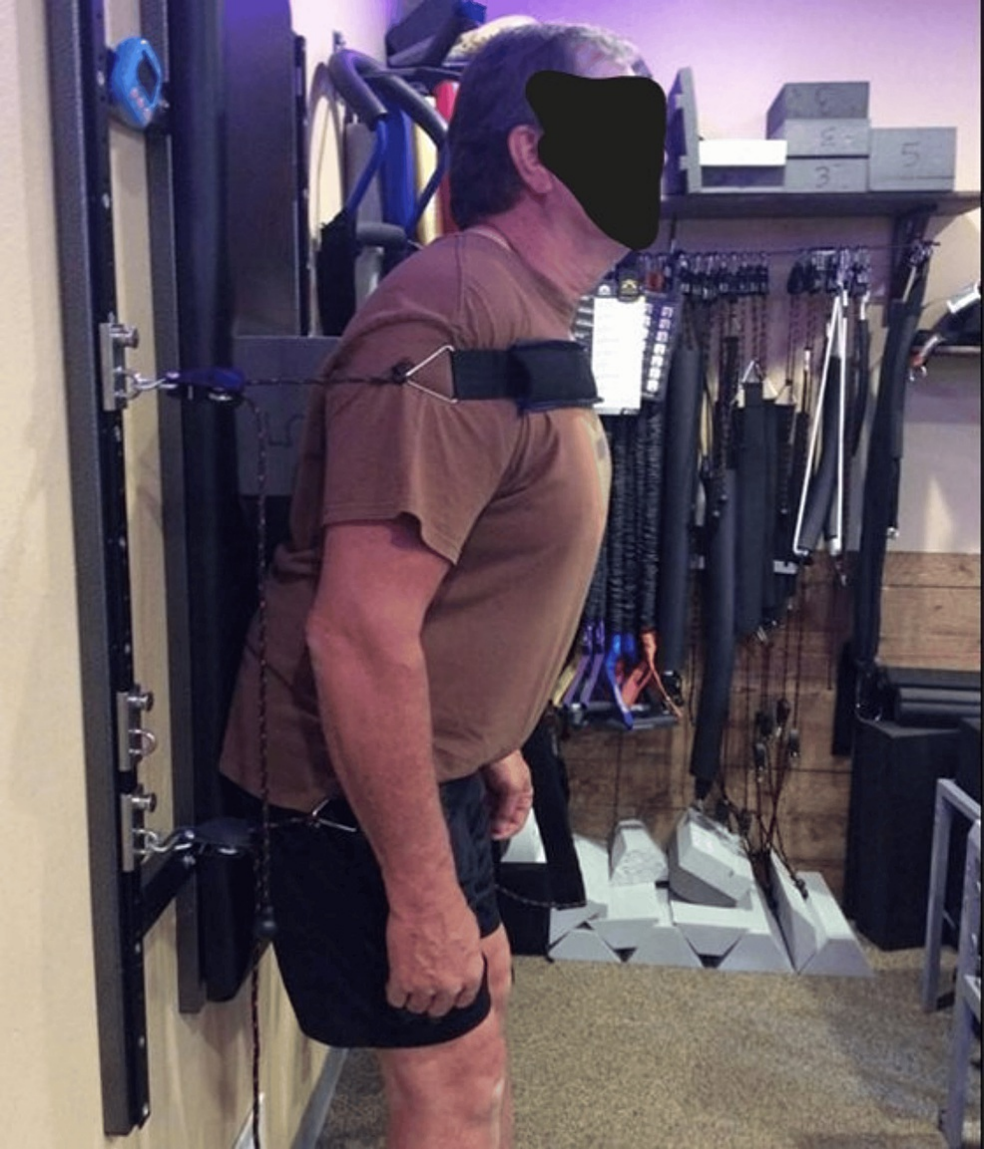
MI® standing traction The patient is standing while a force is translating the thorax anterior, and the pelvis is allowed to flex forward while the patient is instructed to translate the head posterior.

The patient elected to undergo a structural and functional rehabilitation program designed to improve postural muscle strength, decrease sagittal and coronal abnormalities, and improve spine alignment and pain. All PRO’s subjective and objective outcomes would be re-assessed following a period of treatment. Therapy was in-office three times per week for four weeks. Seated MI® traction was performed with a distraction load and a transverse load across the mid-cervical spine to induce lordosis (Figure 1). Meyers cervical remodeling collar (CRCollar™, Circular Traction LLC., Huntington Beach, CA, USA) was utilized to increase lordosis and improve sagittal postures [22]. Left shoulder shrug postural exercise with 10lbs in the left on the whole-body vibration (WBV) device designed by Power Plate® (Power Plate USA, Northbrook, IL, USA) was performed for two minutes per treatment (Figure 3). ProLordotic™ (Circular Traction LLC., Huntington Beach, CA, USA) was used on the Power Plate holding for five seconds with two seconds of rest for two minutes per treatment [22,23]. Additionally, posterior pelvic translation exercises were performed on the Power Plate® to further strengthen postural muscles [24]. The subject received MI® spinal manipulation therapy (SMT) designed to increase abnormal ROM, reduce pain and improve coronal and sagittal posture. MI® traction based upon the measured spinal abnormal radiography was used reduce abnormal spine alignment. The traction involves a force to place the thorax anteriorly, while simultaneously pressing the pelvis posteriorly and having the patient translate their head posterior with simultaneous slight extension (Figure 4). The subject demonstrated good compliance and tolerance to the treatment and the initial treatment plan was completed in accordance with prior studies of CBP® methods followed by re-assessment of initial outcomes [25].

Results

Following 12 treatments, the patient was re-assessed for progress in subjective and objective outcomes. At the time of examination, he reported his LBP, and the primary source of his disability was improved by 90%. His lumbago that was associated with radicular leg pain was resolved. The sexual dysfunction remained. The MBP, thoraco-lumbar and cervicothoracic regional pain was resolved, and the bilateral shoulder and lower neck pain was resolved. Palpation of regional soft tissues found tenderness only at the upper cervical spine on the left and the lower lumbar spine on the left. Cervical compression pain provocation orthopedic testing in neutral was positive for pain on the right cervical spine and was the only positive pain provocation test. This is a stark improvement to the multiple positive compressive and pain provocation tests at initial examination, performed by the same physician (TJW).

All ROM measured for pain provocation visually was found to be within-normal-limits (WNL) and without any reported pain, discomfort or disability. Patellar reflexes remained reduced (R+1, L+2). Some mile hypoesthesia at L5 right dermatome remained. The patient reported a normal level of activities of daily living (ADLs) good quality of life (QOL) and no restrictions in function. He was instructed to continue to perform his prescribed daily postural exercises and Denneroll® (Denneroll® Spinal Orthotics, Wheeler Heights, NSW, Australia) traction and return for in-office for 12 treatments once-per-month due to distance to the facility. He was compliant with the program.

**Figure 5:**
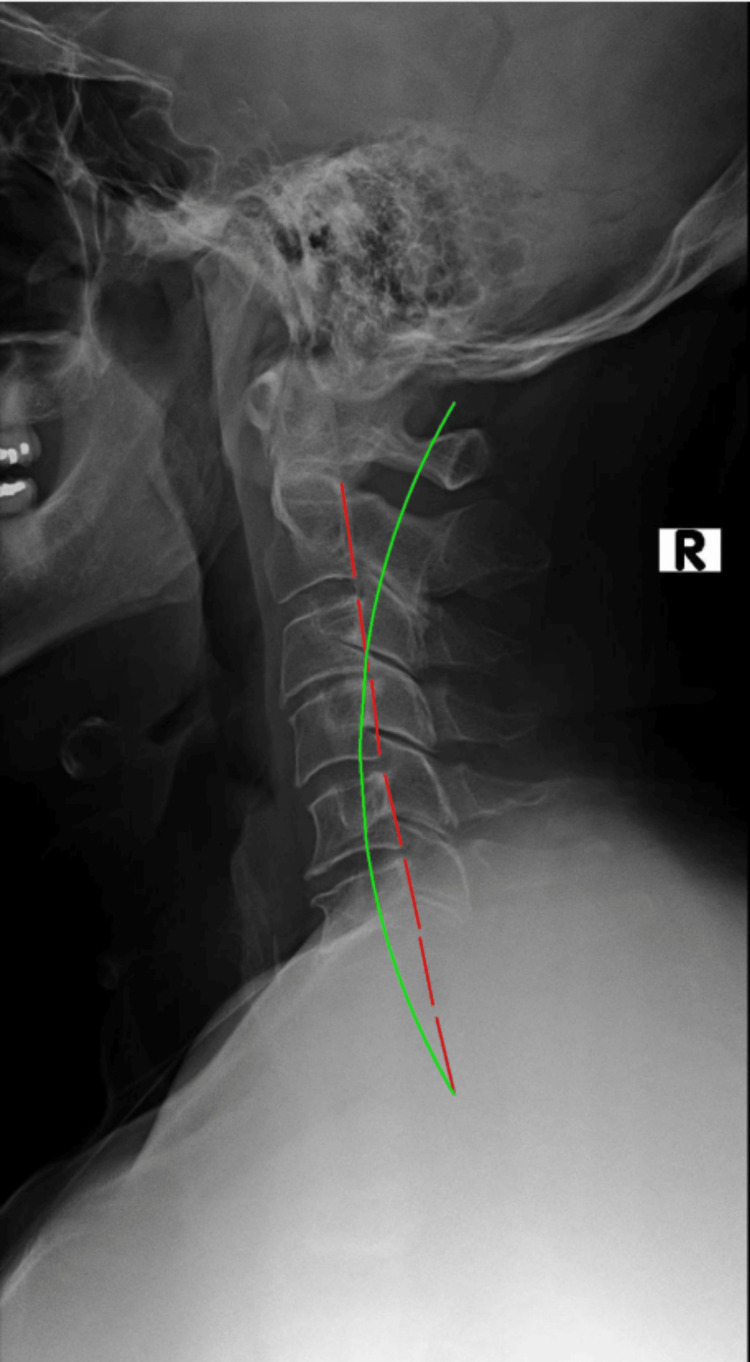
Re-examination lateral cervical radiograph following 12 in office treatments and 24 Denneroll® home treatments The red line demonstrates abnormal spine alignment. The green line represents ideal lateral cervical lordosis.

**Figure 6:**
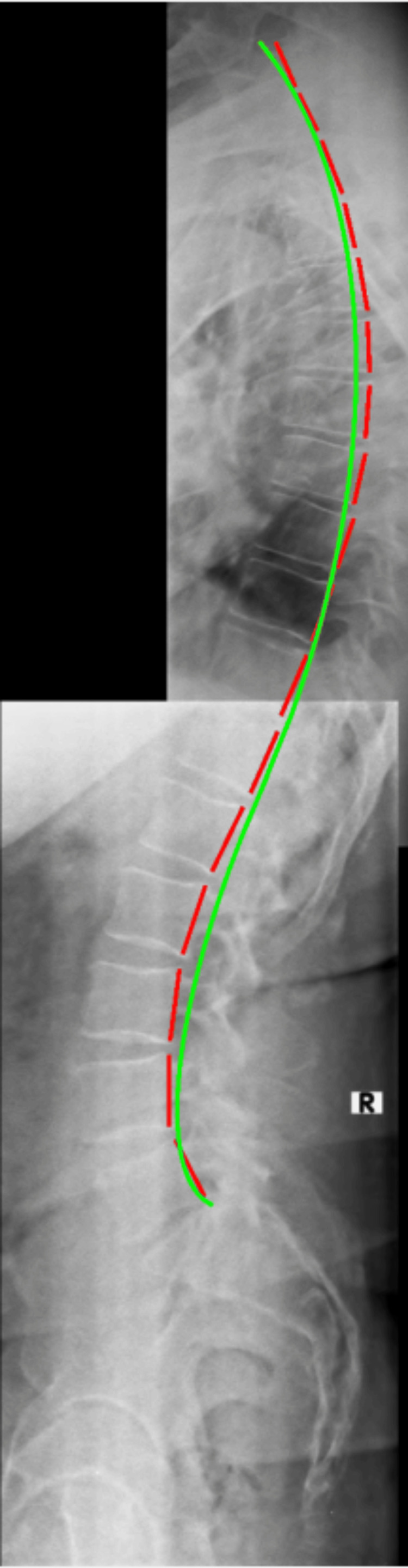
Re-examination lateral thoracic and lateral lumbar radiographs stitched Lateral thoracic and lumbar radiographs demonstrated significant improvement in spine alignment and sagittal balance. The dashed red line measures vertebral body posterior tangents and the green line represents ideal thoracic and lumbar curvature.

Following a year and 24 home treatments with the Denneroll®, the patient returned and symptoms remaining were the headaches (16/100-very mild), NDI (10/100-very mild), QVAS for the cervical spine (4/10 mild-moderate), RODI for the LBP was significantly improved (12/100-mild), QVAS for the MBP was improved (20/100), QVAS for the LBP was greatly improved (23/100-mild), and the other conditions that had reduced his PROs were significantly improved vs. baseline. Re-assessment of all HRQoLs and postural and structural coronal and sagittal balance measures were replicated. SF-36 health status questionnaire was greatly improved with only social function due to pain below normative data (75/100 vs 83/100). Health function restriction (77/100), physical function restriction due to pain (95/100), emotional disability due to pain (100/100) mental health disability due to pain and suffering (80/100) bodily pain impact on disability (78/100), and lack of energy /increased fatigue due to pain and suffering (80/100). All SF-36 measures were improved vs. baseline and above average normative data at one-year follow-up (Table 1). Posture (Figures 5, 6) and radiographic analysis of upright digitized sectional and stitched full-spine films found stability and some continued improvement. Absolute rotation angle (ARA) measured using the Harrison Posterior Tangent Method found the long-term re-examination radiograph had increased lordosis from initial -2.5° to -11.8° which shows MCID is maintained long-term.

**Table 1:** Results of the 36-question health status questionnaire pre-/post-/follow-up The patient demonstrated improved health status across multiple sub-categories at initial re-evaluation and maintained long-term.

Date	Health	Physical	Physical	Emotional	Social	Mental	Bodily	Energy
	Perception	Function			Function	Health	Pain	Fatigue
Normal	72	84	81	81	83	78	75	61
8/11/2020	57	80	50	100	75	84	58	50
9/6/2022	77	95	100	100	75	80	78	80
9/8/2023	87	95	100	100	100	80	90	80
Overall Change	30	15	50	0	25	-4	32	30

The patient was treated 12 more time over the course of a year with in-office MI traction, MI exercise and MI SMT. Home exercises and Denneroll® traction continued.

Three-year re-assessment from initial encounter was performed in September of 2023.The patient continued to report more than 90% improvement in the initial LBP. Leg radicular pain was 80% improved. The sexual dysfunction had resolved, MP was 100% resolved, left shoulder pain was reported 100% resolved and the headaches were significantly improved. HDI demonstrated very mild headache (20/100), NDI found very mild neck pain (6/100) and MODI remained significantly improved (2/50). Cervical QVAS found no pain (0/100). Thoracic QVAS was mild (13/100) and lumbar QVAS found minimal pain (17/100). All measures of the Rand SF-36 were found to be at or above normative data (87/100, 95-100, 100/100, 100/100, 100/100 80/100, 90/100, 80/100) for health perception, physical functioning, physical functioning, role physical, role functioning, social functioning, bodily pain, mental health and energy/fatigue, respectively.

**Figure 7:**
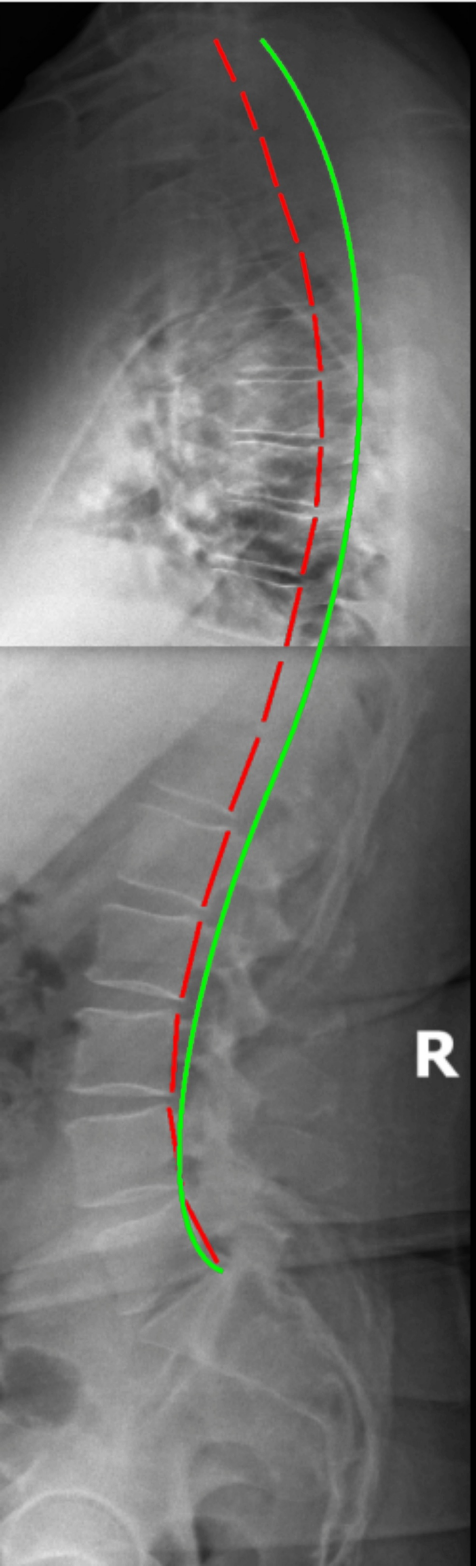
Three-year lateral thoracic and lateral lumbar radiographs demonstrating slight increased forward sagittal balance Long-term stitched lateral lumbar and lateral thoracic show some increase in positive translation of the torso; however, the patient maintained good contours with no in-office treatment for three years.

Radiography showed maintained, WNL translation values in the cervical, thoracic, and lumbar spine, some loss of normal cervical curvature, and some degenerative changes in the mid-cervical segments (C3/C4, C5/C6 and C; T7/T8 and T8/T9; L3/L4 and L5/S1) (Figure 7). However, this degeneration was present in some amount in all of the radiographic assessments and did not appear to be significantly worse at the long-term follow-up. Long-term reduction in spinal pain with maintained sagittal and coronal balance were found both with the visual postural assessment and the radiographic findings. The long-term exam found no significant findings that would contribute to additional disability, apart from degeneration in some segments in all three regions of the spine, but they did not appear to be symptomatic at the time of evaluation. Exam found continued improved spine alignment and the patient was very satisfied with care and his minimal disability for the prior several years. The patient continues to perform home postural exercises and home Denneroll® traction.

## Discussion

The aim of this study is to present the successful treatment in a patient suffering from pain and disability due to long term results of injury as teen. This study can provide clinicians with a treatment option for treating patients suffering with chronic pain. CLBP is the most significant cause of disability globally and the burden for the patient, society, and third parties is tremendous, and LBP contributes the most to years lived with disability (YLD) [26]. The contribution to the global burden of GBD, is compounded when the patient suffers from CWSP including NP, HA, and extremity pain with radiculopathy and hypoesthesia. The likelihood of disability and lost productive years for the individual suffering from multiple spine conditions is indisputable [27]. Reducing CWSP as well as mechanical CLBP with safe, reliable, and economical protocols is a desirable clinical outcome.

Injuries in childhood that are untreated or do not receive adequate treatment to resolve the symptoms and dysfunction most frequently lead to spinal pain as an adult [28]. Our patient reported a repetitive stress injury in a very young adolescent spine, which likely caused pro-inflammatory changes in and around the spine and spinal tissues, and potentially led to the abnormal coronal and sagittal postural imbalances. Forty-eight years of spinal pain from trauma, abnormal spine alignment and other lifestyle and ergonomic contributors likely led to the patients abnormal subjective and objective findings.

The treatment protocols have previously been published in the medical literature and chronic pain conditions have had successive and positive clinical outcomes in short- and long-term studies from single cases to large RCTs. A multi-modal approach to improving spine alignment, abnormal coronal and sagittal balance and posture, as well as improved HRQoLs with CBP® methods has been demonstrated across multiple conditions [8-10,29-34]. Initial CBP® case studies found that the multiple treatment approach may have been a limitation to the study conclusions. Recently, growing reports demonstrate that this approach to improving postural muscles, postural non-contractile tissues like disc and ligament, via MI® traction, as well as postural SMT is a repeatable and reliable approach for spine and some extra-spinal conditions [8-10,29-34]. CWSP and CLBP is such a burden that having the potential of the safe, efficacious, and repeatable methods of CBP® to reduce GBD is desirable for astute clinicians who treat spine pain and extremely beneficial with low potential for poor outcomes for the patient [35,36].

Abnormal spine conditions are so prevalent and such a GBD that correct diagnosis is imperative for correct treatment protocols to produce the best patient-based outcomes. CBP® protocols require proper historical, neurologic, orthopedic and functional testing as well as posture analysis and radiography to ensure that the patient-specific treatment regimen can be prescribed. This thorough diagnostic process will ensure correct differential diagnosis, patient limitations to correction as well as potential multi-disciplinary approaches are found and utilized. Radiography is criterion standard for the proper diagnosis of the cause of the patients pain. PostureRay® analysis allows clinicians to have a safe and easy and reliable way to determine the abnormal spine shape and can thus apply the correct MI exercises, MI® traction and MI® SMT to produce the best results. The addition of home exercises and traction and lifestyle changes can further add to the efficacious nature of the treatment of CWSP and CLBP. Limitations are individual case design and multi-modal application of therapies. RCTs involving injured teens followed into their sixth decade would be very difficult thus drawing conclusions from the pain-reducing and posture improving previous studies of CBP®, as well as case studies such as this report may be necessary for astute clinicals to seek the most appropriate choice of care for the individual patient.

## Conclusions

We present the positive PROs and better HRQoLs following CBP® protocols to lessen abnormal spinal loads, improve ROM for pain provocation, and decrease pain as well as improve sagittal balance, coronal balance, and spine alignment in a patient's suffering for many decades with pain and disability. This case adds to the growing body of evidence demonstrating the efficacious, economical, safe, repeatable, and reliable treatment regimens of CBP®. Given the widespread cross-population effect of chronic spine pain on society and individuals, additional studies and funding are warranted to study the beneficial effects of an inexpensive, repeatable protocol such as CBP®. Further studies are necessary to determine if CBP® reduces GBD.
